# Cognitive complaints in older adults in primary care and associated factors

**DOI:** 10.1590/1980-5764-DN-2022-0096

**Published:** 2023-05-29

**Authors:** EL Mahjoub EL Harsi, Abdelhafid Benksim, Mohamed Cherkaoui

**Affiliations:** 1 Cadi Ayyad University, Faculty of Sciences Semlalia, Laboratory of Pharmacology, Neurobiology, Anthropobiology and Environment, Marrakech, Morocco.; 2 Regional Health Directorate, Higher Institute of Nursing Professions and Health Techniques, Nursing Care Department, Marrakech, Morocco.

**Keywords:** Cognitive Dysfunction, Aged, Memory, Disfunção Cognitiva, Idoso, Memória

## Abstract

Cognitive complaints are common in older people. They can progress into a more severe cognitive decline and then deterioration of quality of life. They are associated with several factors.

**Objective:**

This study aimed to determine the factors associated with cognitive complaints in older adults in the city of Marrakech, Morocco.

**Methods:**

This study was conducted between March and June 2022 among 281 people aged 50 years and older who attended primary health care centers in the city of Marrakech. Cognitive complaints were measured using the McNair-Kahn scale. Data on sociodemographic and clinical characteristics were collected through interviews with the participants and consultation of their medical records. Analysis was done using Statistical Package for Social Sciences (SPSS) version 25, Ink software.

**Results:**

Of the total participants, 51.6% had cognitive complaints. Multivariate analysis showed that people aged 75 years and over had a sevenfold higher risk of cognitive complaints than people aged 50–64 years (p=0.033; OR=7.64; 95%CI 1.17–49.72), and that illiteracy (p=0.004; OR=3.39; 95%CI 1.48–7.76), cardiovascular disease (p=0.018; OR=4.30; 95%CI 1.29–14.32), diabetes (p=0.001; OR=3.14; 95%CI 1.64–6.04), visual impairment (p=0.017; OR=2.22; 95%CI 1.15–4.19), depression (p= 0.027; OR=2.36; 95%CI 1.10–5.05) and sleepiness (p=0.034; OR=1.96; 95%CI 1.05–3.66) are associated variables.

**Conclusions:**

Cognitive complaints are frequent in older adults and are associated with several sociodemographic and health factors. Some measures could help maintain stable memory performance in old age and prevent severe cognitive declines, such as regular follow-up of at-risk individuals, and cognitive, physical and leisure activities.

## RESUMO.

Queixas cognitivas são comuns em pessoas idosas. Elas podem evoluir para um declínio cognitivo mais grave e, em seguida, deterioração da qualidade de vida. Elas estão associadas a vários fatores.

**Objetivo:**

Este estudo teve como objetivo determinar os fatores associados às queixas cognitivas em idosos da cidade de Marrakech, Marrocos.

**Métodos:**

Este estudo foi realizado entre março e junho de 2022 entre 281 pessoas com 50 anos ou mais que se apresentaram em centros de atenção primária à saúde na cidade de Marrakech. As queixas cognitivas foram medidas usando a escala de McNair-Kahn. Os dados sobre características sociodemográficas e clínicas foram coletados por meio de entrevista com os participantes e consulta aos seus prontuários. A análise foi feita usando o *software* Pacote Estatístico para as Ciências Sociais (SPSS), versão 25.

**Resultados:**

Do total de participantes, 51,6% apresentaram queixas cognitivas. A análise multivariada mostrou que pessoas com 75 anos ou mais tiveram risco sete vezes maior de queixas cognitivas do que pessoasentre 50–64 anos (p=0,033; OR=7,64; IC95% 1,17–49,72), e que analfabetismo (p=0,004; OR=3,39; IC95% 1,48–7,76), doenças cardiovasculares (p=0,018; OR=4,30; IC95% 1,29–14,32), diabetes (p=0,001; OR=3,14; IC95% 1,64–6,04), comprometimento visual (p=0,017; OR=2,22; IC95% 1,15–4,19), depressão (p=0,027; OR=2,36; IC95% 1,10–5,05) e sonolência (p=0,034; OR=1,96; IC95% 1,05–3.66) são variáveis associadas.

**Conclusões:**

Queixas cognitivas são frequentes em idosos e estão associadas a diversos fatores sociodemográficos e de saúde. Algumas medidas poderiam ajudar a manter o desempenho estável da memória na velhice e prevenir declínios cognitivos graves, como o acompanhamento regular de indivíduos em risco e atividades cognitivas, físicas e de lazer.

## INTRODUCTION

Complaining about forgetfulness and memory problems can happen to people of all ages. However, in older adults, cognitive complaints (CCs) are of particular importance due to their frequency and the risk of developing other more serious cognitive disorders^1^.

CCs are considered benign or trivial when part of the normal cognitive aging process without severity, but they could become warning signs for other early cognitive disorders. Several studies have shown that individuals with CCs may be at increased risk for developing Alzheimer's disease (AD) and dementia^2–4^.

In addition, the cognitive disorders may be responsible for adverse effects on the quality of life of older people leading to dependence, limitation of daily living activities, anxiety, and social isolation^5–7^.

CCs therefore indicate that older people are at risk of experiencing a decline in cognitive functioning and, consequently, in quality of life. They must be taken very seriously when expressed to a family member or a physician.

In the context of the aging population that the world currently lives, maintaining stable memory performance in advanced age represents a major challenge in public health to keep the quality of life of the older people sane and allow them to age well. It is therefore very interesting to conduct studies on CCs in older adults and to research the associated factors.

Several studies have shown that CCs were significantly associated with age^8^, gender^9^, education and physical illness^10,11^, sleep disturbance^12^, and depression^13^.

Although the topic on memory problems in older people is widely addressed in high-income countries, few studies have been conducted in low- and middle-income countries. Recently Smith et al. conducted a study of cognitive impairment in several countries based on data from the 2002–2004 World Health Survey but excluded Morocco due to a lack of data^13^.

The objective of this study was to determine the factors associated with CCs in older adults in the city of Marrakech, Morocco.

## METHODS

### Ethical consideration

All methods adopted in this study were applied in accordance with the principles stated in the Declaration of Helsinki. The study was approved by the Ethics Committee of the Regional Health Directorate of Marrakech-Safi, Morocco (reference: N 26/2021). Written informed consent was obtained from the participants before the start of the study via an information sheet on the conduct of the survey.

### Data collection

Data collection was conducted at three urban health centers in the city of Marrakech that provide primary health care. The target population consisted of older people attending these health services. The inclusion criteria were people aged 50 years and over, and the exclusion criteria were people with severe psychotic disorders such as delusions, hallucinations, etc. The final sample was set at 281 who were selected through convenience sampling.

Data were collected between March and June 2022 through a face-to-face interview with participants using a guide containing open-ended, closed-ended, and multiple-choice questions, and also through consultation of the participants’ medical records.

CCs were measured using the McNair-Kahn scale 15-item^14,15^. This is a subjective scale that measures the individual's cognitive disability in everyday life. It is a questionnaire scored from 0 to 3 exploring various cognitive domains such as: attention-concentration, language, praxis, delayed recall, orientation towards people, spatiotemporal orientation, and prospective memory. Any person who scores higher than 15 is considered to have a CC.

### Variables and modalities

In addition to the data on the CCs, sociodemographic information were collected through the interview with the participants, namely age, gender, origin (urban or rural), marital status (with or without spouse), education (illiterate: those who cannot read or write; primary level: those who attended primary school; secondary level: those who enrolled in junior high and/or high school; and higher level: those who attended university), health insurance (covered or not covered), income according to current or previous occupation (low: less than 3000 Moroccan Dirham (MD); medium: between 3000 and 6000 MD; and high: above 6000 MD).

Clinical data were collected through the participants’ self-reporting or through consultation of the participants’ medical records, i.e., hearing and visual status, presence of chronic disease such as diabetes, cardiovascular disease, hypertension, endocrine disease, bronchopulmonary disease, etc. Nutritional status was assessed by calculating body mass index (BMI) through weight and height. Besides, data were collected on addictive behavior, namely the consumption of cigarettes, cannabis and alcohol.

Independence was measured using the ADL (activities of daily life) scale with three categories:

- Independent: 6–5 score;
- Partially dependent: 4–2 score; and
- Totally dependent: <2 score^16^.

Depression was assessed using the 15-item Geriatric Depression Scale (GDS) ranging from 0 to 15 points:

- Normal: 0–5;
- Mild to moderate depression: 6–9; and
- Severe depression: 10 and more^17^.

The Epworth sleepiness scale was used to look for sleep disorders in the participants. It allows to distinguish four levels of sleepiness according to the score obtained:

- Sufficient sleep: score 0–6;
- Sleep quality that can be improved: score 7–8;
- Excessive daytime sleepiness,
- Probable pathology: score 9–14; and
- Very important daytime sleepiness: score 15 and more.

Thus, any person with a score higher than 8 is considered to have a sleep disorder^18^.

### Statistical analysis

All data were analyzed using the Statistical Package for Social Sciences (SPSS) version 25, Ink software. The dependent variable “cognitive complaints” was coded as 0 (yes), and 1 (no). The association between the categorical variables was investigated with the chi-square test. Student's *t*-test was used to compare the means. In order to consider the different confounding factors and highlight the importance of each of the selected explanatory variables on the dependent variable “cognitive complaints”, multiple logistic regression was used, with the stepwise method and the odds ratio (OR) statistic at 95% confidence interval (95%CI), analyzing any independent variable with which there was a correlation or a statistically significant association. Statistical significance was defined as p-value<0.05.

## RESULTS

Table 1 summarizes the sociodemographic characteristics of the participants. A total of 281 people were included in this study, 57.7% were women, 63.7% were of urban origin, 51.6% had CCs, and 48.4% had no memory problems. The average age of those with CCs was 68.76±10.12, and the ones without CCs was 62.53±8.91.

**Table 1:** Sociodemographic characteristics of participants with and without cognitive complaints.

Variables		Cognitive complaints		p-value
		Yes=145	No=136	
Age (±SD)		68.76±10.120	62.53±8.913	0.001
Age group (years)				
	50 to 64	55 (37.9)	84 (61.8)	0.001
	65 to 74	56 (38.6)	36 (26.5)	
	>75	34 (23.4)	16 (11.8)	
Gender				
	Female	81 (55.9)	81 (59.6)	0.531
	Male	64 (44.1)	55 (40.4)	
Origin				
	Urban	96 (66.2)	83 (61.0)	0.367
	Rural	53 (39.0)	53 (39.0)	
Marital status				
	With spouse	76 (52.4)	81 (59.6)	0.228
	Without spouse	69 (47.6)	55 (40.4)	
Education status				
	Illiterate	65 (44.8)	38 (27.9)	0.021
	Primary	27 (18.6)	35 (25.7)	
	Secondary	48 (33.1)	53 (39.0)	
	Superior	5 (3.4)	10 (7.4)	
Income				
	Low	121 (83.4)	97 (71.3)	0.015
	Medium	23 (15.9)	32 (23.5)	
	High	1 (0.7)	7 (5.1)	
Health insurance				
	Covered	88 (60.7)	91 (66.9)	0.278
	Not covered	57 (39.3)	45 (33.1)	

Abbreviations: SD: standard deviation. Note: (): absolute frequency.

More people with CCs reported being illiterate, compared to the ones without CCs (44.8 vs. 27.9%), and that they were low-income (83.4 vs. 71.3%). No significant difference was found between the two groups in relation to medical coverage.

Table 2 presents the health characteristics and self-reported morbidities among the participants. This table shows that compared to people without CCs, those who claimed CC had more cardiovascular conditions (16.6 vs. 3.7%), hypertension (44.1 vs. 29.4%), diabetes (62.1 vs. 41.9%), hearing loss (40 vs. 20.6%), and visual impairment (64.8 vs. 38.2%). There was no significant difference between the two groups regarding nutritional status (p³0.05).

**Table 2:** Health characteristics and self-reported morbidities in older adults with and without cognitive complaints.

Variables		Cognitive complaints		p-value
		Yes=145	No=136	
Cardiovascular conditions		24 (16.6)	5 (3.7)	0.001
Hypertension		64 (44.1)	40 (29.4)	0.011
Diabetes		90 (62.1)	57 (41.9)	0.001
Hearing loss		58 (40.0)	28 (20.6)	0.001
Vision disorder		94 (64.8)	52 (38.2)	0.001
Nutritional status: BMI (kg/m^2^)				
	Undernutrition	7 (4.8)	1 (0.7)	0.146
	Normal	59 (40.7)	56 (41.2)	
	Overweight	50 (34.5)	56 (41.2)	
	Obesity	29 (20.0)	23 (16.9)	
Activities of daily living				
	Independent	103 (71.0)	123 (90.4)	0.001
	Partially dependent	34 (23.4)	13 (9.6)	
	Totally dependent	8 (5.5)	0 (0.0%)	
GDS (short form)				
	Normal	67 (46.2)	107 (78.7)	0.001
	Moderate depression	40 (27.6)	19 (14.0)	
	Severe depression	38 (26.2)	10 (7.4)	
	Sleepiness	72 (49.7)	38 (27.9)	0.001
Addiction				
	Cigarettes	36 (24.8)	24 (17.6)	0.142
	Cannabis	20 (13.8)	9 (6.6)	0.048
	Alcohol	21 (14.5)	11 (8.1)	0.092

Abbreviations: BMI: body mass index; GDS: Geriatric Depression Scale.

The assessment of ADL showed that partial dependence was detected more in people who reported CCs than in people without CCs (23.4 vs. 9.6%), whereas total dependence was present only in people with CCs (5.5%).

Similarly, the analysis of the geriatric depression scale showed that more people with CCs had moderate depression (27.6%) compared to those without CCs (14.0%), and the same happened with severe depression (26.2 vs. 7.4%), respectively.

Also, the assessment of sleep quality showed that sleepiness was reported more by people with cognitive impairments (49.7 vs. 27.9%).

As for addiction, the analysis showed that there was no significant relationship between CCs and tobacco and alcohol use; however, cannabis use was reported more by people with CCs (13.8 vs. 6.6%).

Table 3 shows the independent variables significantly associated with CCs according to the multiple logistic regression model. This analysis revealed that people aged 75 years and older are seven times more likely to suffer from CCs than those aged 50–64 (p=0.033; OR=7.64; 95%CI 1.17–49.72). In addition, multiple logistic regression found that illiteracy (p=0.004; OR=3.39; 95%CI 1.48–7.76), cardiovascular diseases (p=0.018; OR=4.30; 95%CI 1.29–14.32), diabetes (p=0.001; OR=3.14; 95%CI 1.64–6.04), visual impairment (p=0.017; OR=2.22; 95%CI 1.15–4.19), depression (p=0.027; OR=2.36; 95%CI 1.10–5.05), and sleepiness (p=0.034; OR=1.96; 95%CI 1.05–3.66) are variables associated with CCs.

**Table 3:** Variables independently associated with cognitive complaints according to the multiple logistic regression model.

Variables		β	Wald	p-value	OR (95%CI)
Age (years)		Reference category			
	50 to 64				
	65 to 74	0.782	1.936	0.164	2.19 (0.73–6.57)
	>75	2.033	4.521	0.033	7.64 (1.17–49.72)
Illiterate		1.221	8.368	0.004	3.39 (1.48–7.76)
Diabetes		1.145	11.812	0.001	3.14 (1.64–6.04)
Cardiovascular diseases		1.458	5.642	0.018	4.30 (1.29–14.32)
Visual disorders		0.787	5.703	0.017	2.22 (1.15–4.19)
Depression		0.858	4.871	0.027	2.36 (1.10–5.05)
Sleepiness		0.674	4.508	0.034	1.96 (1.05–3.66)

Abbreviations: OR: odds ratio; CI: confidence interval.

Notes: β: coefficient; p: significance level of Wald test.

## DISCUSSION

The present study involved 281 individuals aged 50 years and older, 145 of whom had CCs. Analysis of the results showed that CCs are associated with several sociodemographic, clinical and psychological factors.

The results showed that people aged 75 years and older are seven times more likely to have CCs than people aged 50–64. This result is consistent with several research papers which found that the frequency of CCs increases with age and older people are more likely to have CCs than younger subjects^8,19^. Some studies claimed that brain aging leads to changes in white matter morphology and brain function, which promote CCs^19^. However, others found no strong association between age and CCs^20,21^; they explained this inconsistency by the different age categories included in the studies and by the difference between young and old individuals regarding their perceived memory abilities. The papers stated that younger participants relate their CCs to reversible and manageable causes such as stress and concentration problems, allowing them to report good memory more than older subjects who are convinced that their CCs are related to aging, which is irreversible^22^.

Our results also showed that illiteracy is strongly associated with CCs, which is in agreement with several studies that have announced that the percentage of CC is inversely related to education level^10,23^. Authors explained this postulate by the cognitive reserve that educated people can have and that makes a difference in the brain structure and in the execution spots, which allows individuals with higher levels of education to be more resistant to age-related cerebral alterations^24,25^.

This study did not find an association between gender and CC, which is also consistent with several other studies^26,27^. In contrast, some works stated that women are more likely to have memory disorders than men due to changes in sex hormones in menopause^28,29^. This inconsistency in results was explained in some studies, which proved that the difference in memory CC between the sexes is caused by the presence of several other risk factors for cognitive impairment^30^.

With regard to clinical characteristics, the analysis revealed that diabetes and cardiovascular disease are closely associated with CCs. This result corroborates several papers that explained this association by identifying these diseases as risk factors for cerebrovascular pathologies, which, in turn, constitute a factor favoring cognitive disorders^11,31^.

The results of our study also raised an association between vision deficit and CCs which are in accordance with diverse studies that mentioned that older people with vision impairment are more likely to have memory disorders^32,33^. Some have justified this association by the reduction in mental activity caused by this deficiency, stating that the visual deficit may result in a prolonged lack of necessary sensory input, which promotes neural atrophy and may, in turn, cause cognitive deterioration^34,35^.

In addition, depression was identified through our study as a factor independently associated with CCs, which is consistent with several studies that announced that this association could be bidirectional. They stated that oxidative and nitrosative stress related to depression contributes to neurodegeneration and may promote memory problems; they also raised that, on the other hand, the forgetfulness that people with CCs have can trigger depressive symptoms in them^13,36,37^.

Our results also revealed that drowsiness was strongly associated with CCs which corroborates various studies that showed that regular sleep in older adults improves their learning, memory, and information reactivation abilities^12,38^.

Finally, this study has some limitations since the survey had a small sample size and the collection of some data related to health status was based on self-report and perception of the participants. Despite these limitations, this work could provide a database for several future papers on cognitive impairment in older adults in low- and middle-income countries where research on this topic remains limited.

In conclusion, CCs are common in older people and represent an increased risk of deterioration in cognitive ability and therefore in their quality of life. This study found that CCs are associated with several factors, namely advanced age, illiteracy, diabetes, cardiovascular disease, vision problems, sleepiness, and depression. It is very important to pay attention to the presence of these factors in older adults and take the necessary preventive measures to maintain stable cognitive abilities in old age so as to prevent more severe cognitive declines. Several activities can be suggested for the prevention of cognitive impairment, including regular follow-up of chronic diseases, cognitive activities such as number and word games, learning and memory activities, good sleep, physical activities such as walking and yoga, and leisure and entertainment activities.
